# Mechanistic Insight into CM_18_-Tat_11_ Peptide Membrane-Perturbing Action by Whole-Cell Patch-Clamp Recording

**DOI:** 10.3390/molecules19079228

**Published:** 2014-07-02

**Authors:** Anna Fasoli, Fabrizio Salomone, Mascia Benedusi, Claudia Boccardi, Giorgio Rispoli, Fabio Beltram, Francesco Cardarelli

**Affiliations:** 1 Dipartimento di Scienze della Vita e Biotecnologie, Università di Ferrara, Via L. Borsari 46, I-44100 Ferrara, Italy; 2 NEST, Scuola Normale Superiore and Istituto Nanoscienze-CNR, Piazza San Silvestro 12 - 56127 Pisa, Italy; 3 Center for Nanotechnology Innovation @NEST, Istituto Italiano di Tecnologia, Piazza San Silvestro 12 - 56127 Pisa, Italy

**Keywords:** CPP, AMP, chimera, patch-clamp, toroidal-pore, carpet

## Abstract

The membrane-destabilization properties of the recently-introduced endosomolytic CM_18_-Tat_11_ hybrid peptide (KWKLFKKIGAVLKVLTTG-YGRKKRRQRRR, residues 1–7 of cecropin-A, 2–12 of melittin, and 47–57 of HIV-1 Tat protein) are investigated in CHO-K1 cells by using the whole-cell configuration of the patch-clamp technique. CM_18_-Tat_11_, CM_18_, and Tat_11_ peptides are administered to the cell membrane with a computer-controlled micro-perfusion system. CM_18_-Tat_11_ induces irreversible cell-membrane permeabilization at concentrations (≥4 µM) at which CM_18_ triggers transient pore formation, and Tat_11_ does not affect membrane integrity. We argue that the addition of the Tat_11_ module to CM_18_ is able to trigger a shift in the mechanism of membrane destabilization from “toroidal” to “carpet”, promoting a detergent-like membrane disruption. Collectively, these results rationalize previous observations on CM_18_-Tat_11_ delivery properties that we believe can guide the engineering of new modular peptides tailored to specific cargo-delivery applications.

## 1. Introduction

The successful delivery of cargo-molecules into cells can be based on constitutive endocytic processes by exploiting vectors that can ensure: (*i*) high-yield, non-toxic cellular uptake of the cargo moiety, and (*ii*) efficient endosomal escape of the cargo in order to let it reach its specific target. In recent reports by some of us [1,2,3], these properties were conferred to a novel chimeric peptide in which Tat_11_ arginine-rich motif (YGRKKRRQRRR, residues 47–57 of HIV-1 Tat protein, a classical cell-penetrating peptide, CPP) [4] is fused to CM_18_, a membrane-perturbing amphipathic α-helical sequence derived from the cecropin-A/melittin hybrid antimicrobial peptides (AMPs) series (KWKLFKKIGAVLKVLTTG, residues 1–7 of cecropin-A and 2–12 of melittin) [5,6]. CM_18_-Tat_11_ was exploited to promote the cytosolic delivery of a vast range of co-administered cargoes (calcein, GFP, dextrans) [1], and to afford efficient transfection of complexed DNA plasmids [2,3]. CM_18_-Tat_11_ was shown to retain both Tat_11_ ability to enter eukaryotic cells by endocytosis [7] and CM_18_ membrane-perturbing properties. Yet, the mechanism through which this chimera actually alters endosomal-membrane integrity in eukaryotic cells is still unknown. Since CM_18_-Tat_11_ does contain a functional antimicrobial peptide (CM_18_), it is conceivable that it may act by one of the three “canonical” mechanisms of membrane permeabilization proposed for this class of sequences, *i.e*., the “barrel-stave”, the “toroidal”, and the “carpet” model (reviewed in [8,9]). Briefly, in the barrel-stave mechanism the peptide monomers aggregate (as staves) around a central lumen, forming a pore (the barrel): the peptide hydrophobic segments align with the lipid core region of the bilayer, while their hydrophilic segments face the pore lumen. In the toroidal-pore mechanism, the polar segments of the peptides associate instead with the polar head groups of the lipids, so that the lipids are forced to tilt up to form a continuous bend from one side to the other of the membrane. Finally, in the carpet mechanism, the strong electrostatic interaction between the peptides and the phospholipid head groups lead to a peptide-induced membrane ‘carpeting’ effect. Eventually, the peptides can form micelles, thus leading to bilayer disintegration in a detergent-like manner. The possibility to probe these mechanisms directly on eukaryotic-cell membranes would be paramount to understand the mode of action of CM_18_-Tat_11_. To this end, an experimental platform has been recently proposed by some of us, consisting in the whole-cell voltage-clamp analysis of currents on the plasma membrane of rod outer segments isolated from frog retinae, where endogenous conductance can be fully blocked by bright light [10,11,12,13]. By this approach the membrane permeabilization properties of various synthetic or naturally occurring peptides were investigated, and a rationale was proposed to distinguish between the “barrel-stave”, the “toroidal-pore”, and the “carpet” mechanisms, as follows. The barrel-stave model requires that a certain number of monomers bind together once in the plasma membrane to form an ion conductive pore. If the number of monomers inserted in the membrane is small, as it occurs at low concentrations of a peptide like alamethicin, it is expected that the pores form and disaggregate frequently, producing sustained single channel events. The application of larger peptide concentrations is expected to produce macroscopic currents that recover to *zero* upon extracellular peptide removal, because the interaction between the peptide monomers and the membrane is not strong enough to keep the peptides stably inserted once the extracellular supply is ceased. Consequently, the kinetics of current activation and deactivation and its amplitude are expected to be constant at each peptide application. All these features were observed with alamethicin F50/5 [9,10], and are therefore considered in the following as a “signature” of the barrel-stave permeabilization process. In the case of the toroidal model, the molecular attractive forces between the polar head groups of the lipids and peptides are such that the lipids are forced to tilt up and form pores whose walls are constituted by both lipids and peptide monomers. Such a strong interaction, however, is not expected to produce rapid pore formation and disaggregation at low peptide concentrations (*i.e*., sustained single channel events), while larger peptide concentrations are expected to produce macroscopic currents with a *‘delay’* (see Experimental Section), and time constants of current activation (*τ_a_*) and recovery (deactivation; *τ_d_*) larger than in the case of the barrel-stave process. This is because of the longer time needed to form pores whose walls are not composed just by peptide monomers but by tilted lipids and peptides, and because of the longer time to disassemble this stable structure when extracellular peptide supply is ceased. If the peptide application is short and at low concentration, the monomers left on the membrane after peptide removal do not have a concentration large enough to give rise to conductive pores: these peptides may yet contribute to form additional pores once the peptide is applied again extracellularly. Therefore, repeated peptide applications are expected to elicit currents that only initially recover to the *zero* level upon peptide removal, and then produce a progressive acceleration of the current-activation kinetics (*i.e*., decrease of *delay* and *τ_a_*), and a progressive increase of the steady-state current amplitude. This would eventually lead to the formation of stable conductive channels, which would produce progressively larger background currents upon peptide removal. All these features were observed with CM_15_ [11], a peptide well-known to form pores according to the toroidal model [13], and are therefore considered in the following as a “signature” of this particular permeabilization process. Finally, the “signature” of the carpet mechanism is given by two main observables: (*i*) a larger *delay* with respect to the one characterizing current activation in the toroidal and barrel-stave mechanisms (due to the expected slow process of micelle formation); and (*ii*) the substantial irreversibility of the permeabilization process, due to the membrane disruption in a detergent-like fashion, as observed in the case of viroporins [12].

In the following, we have applied the above approach to investigate the permeabilization mechanism of CM_18_-Tat_11_ (and of isolated modules) inserted in the plasma membrane of single CHO-K1 cells. We find that the addition of the Tat_11_ module to CM_18_ is able to trigger a shift in the mechanism of membrane destabilization from “toroidal” to “carpet”, promoting a detergent-like membrane disruption. Collectively, these results rationalize our previous observations on CM_18_-Tat_11_ delivery properties.

## 2. Results and Discussion

Our goal here is to employ the patch-clamp technique to study the mechanism of membrane permeabilization induced by the pore-forming peptides, under strict physiological conditions. This goal is achieved by recording the ion current through the channels formed by these peptides, once inserted in a cell plasma membrane. To avoid contamination by the cell membrane currents, all the endogenous current sources must be blocked, possibly without using any drug (such as TTX, TEA, dihydropyridines, *etc*.) that could obstruct the peptide pores or interfere with the pore formation. We previously have found that the photoreceptor rod outer segment, mechanically isolated from the frog retina, is a suitable preparation because its sole membrane conductance can be fully closed by bright light [9]. In line with our previous reports on CM_18_-Tat_11_ [1,2], we used the CHO-K1 cell line as case study. Nicely, we found that, in symmetric ionic conditions, CHO-K1 cells have no voltage- and/or calcium- and/or time-dependent conductances, but only a very small background one.

**Figure 1:**
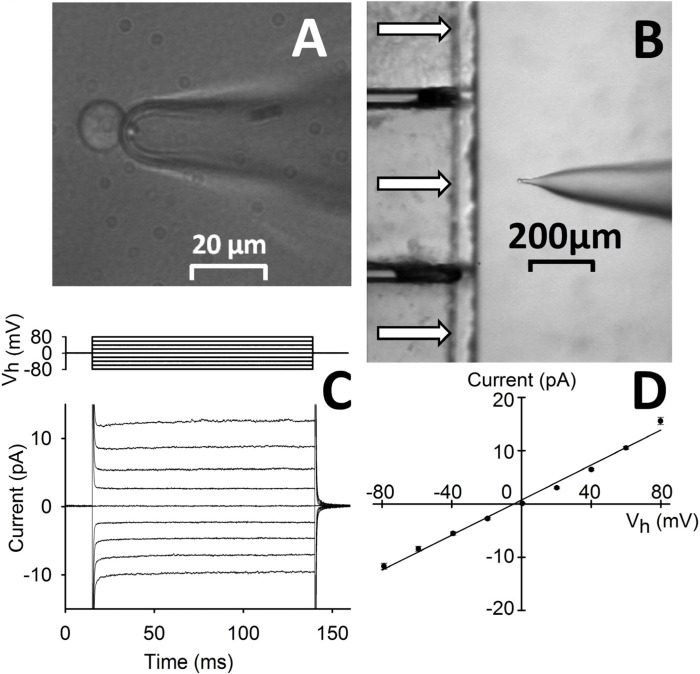
Outline of the technique employed. (**A**) CHO-K1 cell recorded in whole-cell with a pressure-polished pipette; cell is aligned in front of the perfusion pipette (at low magnification in (**B**) formed by square glass capillaries (500 µm of side) glued together; horizontal arrows denote perfusion flows. (**C**) Average whole-cell current recorded from representative cells (*lower panel*; pipette and external solution: 130 mM K^+^ + 1 mM Ca^2+^), subjected to 125 ms voltage steps from −80 mV to +80 mV in 20 mV increments (*top panel*) starting from *V_h_* = 0 mV (traces are the average of *n* = 6 cells); (**D**) the average current evoked by each voltage step of **C** is plotted against the voltage step amplitude; the points are well fitted by a straight line (correlation coefficient ~0.99), whose angular coefficient gave *R_m_* ~6.1 GΩ.

**Figure 2:**
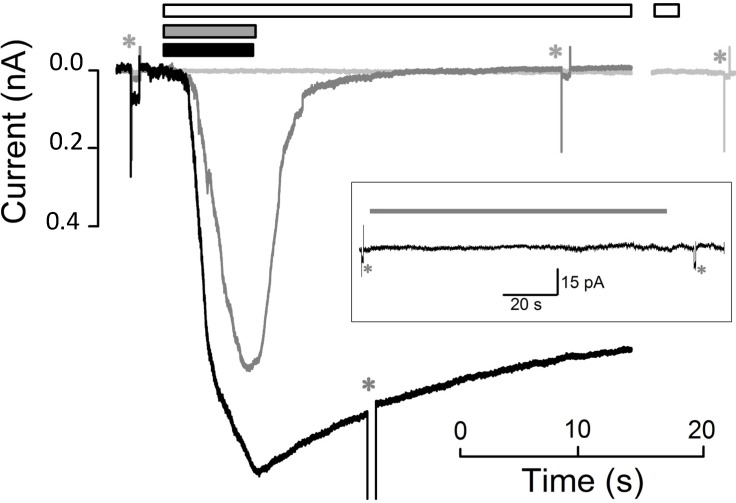
Kinetics of peptide-induced membrane permeabilization of CHO-K1 cells. Whole-cell currents recording elicited by the application (in three different cells), of CM_18_-Tat_11_ (6.7 s, 4 µM; *black* bar and *black* trace), CM_18_ (7.0 s, *grey* bar and *grey* trace), and Tat_11_ (71 s, 8 µM; *white* bar and *light grey* trace; trace break corresponds to 33 s of uninterrupted Tat_11_ perfusion); traces were aligned with peptide timing application; *V_h_* = −20 mV. Inset, 113 s application of 3 µM of CM_18_-Tat_11_; *R_m_* was 1.3 GΩ before and 1.0 GΩ after CM_18_-Tat_11_ application. Asterisks indicate −10 mV pulse delivery, used to measure *R_m_*.

**Figure 3:**
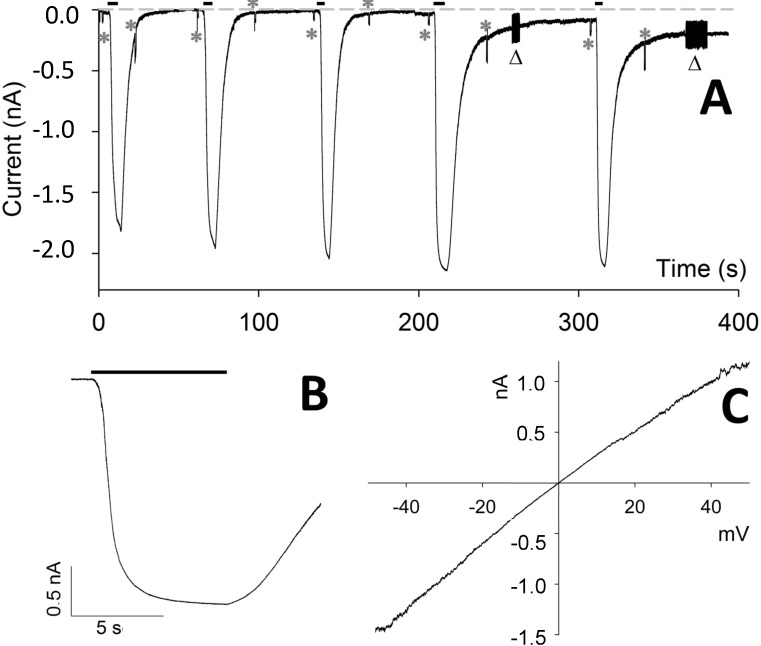
Repeated applications of CM_18_ on CHO-K1 cells. (**A**) Whole-cell current recording elicited by five consecutive applications and withdrawals of 4 µM of CM_18_ lasting, in sequence, 6.1, 5.2, 4.7, 6.2, and 4.1 s; the parameters characterizing the current following each one of the five peptide applications were respectively: *delay*: 1.7, 1.0, 0.9, 0.9, 0.9 s; *τ_a_*: 1.5, 1.2, 0.8, 0.7, 0.7 s; current amplitude: 1.8, 1.9, 2.0, 2.1, 2.0 nA; *τ_d_*: 4.3, 4.4, 4.3, 6.0, 5.2 s. (**B**) A detailed view of the fourth peptide application. (**C**) Voltage dependence of the current elicited by 2 µM CM_18_ corrected for the leakage (in a cell different than **A**). In **A** and **B**: peptide application is indicated by the black bar; *R_m_*, *R_a_*, and membrane capacitance were checked before and after each peptide application by means of a single −10 mV pulse, indicated by an asterisk, and by repeated 10 mV pulses, indicated by a triangle; the grey dotted line indicates *zero* current; *V_h_* = −20 mV.

**Figure 4:**
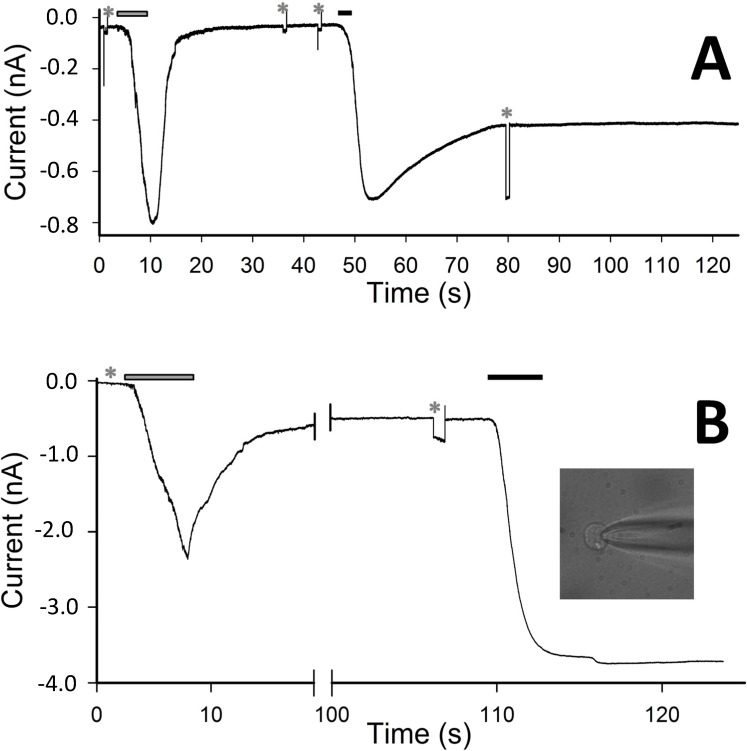
(**A**) Current elicited by the application (for 7 s) and withdrawal of 4 µM CM_18_ (*grey* bar) and of 4 µM CM_18_-Tat_11_ (3 s, *black* bar) on the same cell. (**B**) Whole-cell current recording elicited by the application and withdrawal of CM_18_ (9 s, 8 µM; *grey* bar) and of CM_18_-Tat_11_ (7 s, 8 µM, *black* bar) on the same cell; *inset*, lysis and death of cell following >10 min application of CM_18_-Tat_11_. *R_m_* in **A** and **B** was checked by means of −10 mV pulses, indicated by the asterisks, *i.e*., before CM_18_ application, after CM_18_ withdrawal (and before CM_18_-Tat_11_ application) and after CM_18_-Tat_11_ application, respectively, and found to be: 0.6 GΩ, 0.6 GΩ, 35 MΩ in **A** and 0.6 GΩ, 110 MΩ, and not measurable (too small) in **B**; *V_h_* = −20 mV.

Indeed, the current amplitude is time-independent and very small for any physiological voltage value (Figure 1C). We observe a linear (ohmic) current-to-voltage behavior (Figure 1D) and a membrane resistance (*R_m_*) > 5 GΩ. Such high *R_m_* values make it possible to measure current amplitudes as small as 1 pA in a bandwidth of at least 1 kHz (*i.e*., any exogenous peptide-induced current can be detected down to the single-channel level). Thanks to this high recording resolution, we set *V_h_* = −20 mV in order to limit the current amplitude induced by the strongly permeabilizing peptides while ensuring a detectable current through the weaker ones. In a characteristics measurement, isolated CHO-K1 are held at *V_h_* and *R_m_* is checked before peptide delivery by means of a brief −10 mV step (Figure 2, Figure 3 and Figure 4); various concentrations of CM_18_-Tat_11_ (or CM_18_, or Tat_11_) are then delivered using the fast-perfusion system. Once current stabilizes, the cell is returned to the control solution (without peptide) to assess the possible current level recovery and *R_m_* is again measured. In the control solution, repetitive 10 mV pulses are routinely applied to check access resistance (*R_a_*) stability (Figure 3), otherwise the recording is terminated.

CM_18_-Tat_11_ continuously applied for more than 1 min at various concentrations (0.5 µM, *n* = 2; 1 µM, *n* = 3; 2 µM, *n* = 4; 3 µM, *n* = 5) fails to elicit detectable currents down to single-channel events. A typical example is shown in Figure 2, *inset*, in which the current does not significantly deviate from the *zero* level following a ~100 s application of 3 µM CM_18_-Tat_11_. On the contrary, application of 4 µM CM_18_-Tat_11_ elicits a current that develops in a roughly exponential trend (*τ_a_* = 1.5 ± 0.4 s) up to a steady-state amplitude of 1.0 ± 0.2 nA (*n* = 15 cells) with a *delay* of 2.6 ± 0.5 s (see Experimental Section). As shown by the representative curve in Figure 2 (*black* trace), the induced membrane permeabilization is extensive, as the evoked current does not fully recover to the baseline even after several seconds from peptide removal (see Figure 4 for more details): these recordings are very similar to the ones obtained with a viroporin-derived peptide [13], acting in a carpet-like fashion. Given the structural/functional modularity of CM_18_-Tat_11_, we used the two isolated CM_18_ and Tat_11_ peptides as controls for the observed behavior. CHO-K1 cells exposed to CM_18_ concentrations from 0.5 to 4 µM invariably show detectable membrane currents, *i.e*., membrane destabilization (4 µM: steady state amplitude = 1.0 ± 0.1 nA, *delay* = 0.9 ± 0.2 s, *τ_a_* = 2.1 ± 0.7 s, *τ_d_* =3. 7 ± 1.0 s, *n* = 7 cells; 2 µM: steady state amplitude = 0.36 ± 0.07 nA, *delay* = 3.6 ± 1.1 s, *τ_a_* = 4.0 ± 1.1 s, *τ_d_* = 3.2 ± 1.0 s, *n* = 6 cells; concentrations < 2 µM yield activation and deactivation currents that cannot be simply fitted with a single exponential: statistics are therefore not reported). Contrary to what was found for CM_18_-Tat_11_, however, in this case permeabilization is fully reversible for all tested concentrations upon peptide removal, *i.e*., current returns roughly exponentially to 0, and *R_m_* fully recovers to its former level (representative curve in Figure 2, *dark grey* trace).

However, for repetitive CM_18_ applications at 4 µM (Figure 3A), the recovery of current and of *R_m_* was progressively more incomplete; moreover, the current-to-voltage relationship was found to be linear (a representative recording is illustrated in Figure 3C), showing that the pore formation is voltage-insensitive (differently from other pore-forming peptides, as alamethicin). This relationship was obtained by applying voltage ramps (from −50 to +50 mV, slope: 0.25 mV/ms) during CM_18_ perfusion at V_h_ = −20 mV. To avoid the loss of voltage control due to *R_a_* at extreme voltages (−50 and +50 mV, where currents may become very large), CM_18_ was applied at 2 µM concentration, corresponding to a current not exceeding 2 nA at extreme voltages. The responses to three consecutive voltage ramps during CM_18_ perfusion were averaged and corrected by subtracting the average response recorded in control conditions (Figure 1D and Figure 3C). The obtained relationship is almost perfectly ohmic at physiological voltages, and in all the cells examined (*n* = 4). Notably, all the recordings obtained with CM_18_ were very similar to the ones obtained with the analogous cecropin-A/melittin CM_15_ hybrid peptide, inserted in the plasma membrane of isolated photoreceptor rod outer segments [12] and in CHO-K1 cells (data not shown). Since CM_15_ forms transient toroidal pores in the membrane, as previously demonstrated by patch-clamp analysis [12] and site-directed spin-labeling electron-paramagnetic-resonance studies [14], it is concluded that these two variants share the same membrane-destabilization mechanism. Finally, continuous application of Tat_11_ (up to 5 min) at concentrations from 0.5 to 8 µM failed to elicit any current for all voltage values tested (from −80 mV to +80 mV), thus demonstrating that this module alone is not able to significantly permeabilize the membrane (Figure 2, *light grey* trace). In order to provide a direct comparison between membrane-destabilization properties of CM_18_-Tat_11_ and CM_18_, we recorded data during sequential administration of these peptides to the same cell at 4 µM (Figure 4A). As expected, CM_18_ produces a reversible current, while CM_18_-Tat_11_ leads to irreversible membrane destabilization of the same cell. Increasing the peptide concentration to 8 µM still produces a reversible effect in the case of CM_18_ (although with an incomplete recovery, Figure 4B) while cell lysis and death are the outcomes in the case of CM_18_-Tat_11_ (Figure 4B, and 4B inset, experiment performed on the same cell).

**Figure 5:**
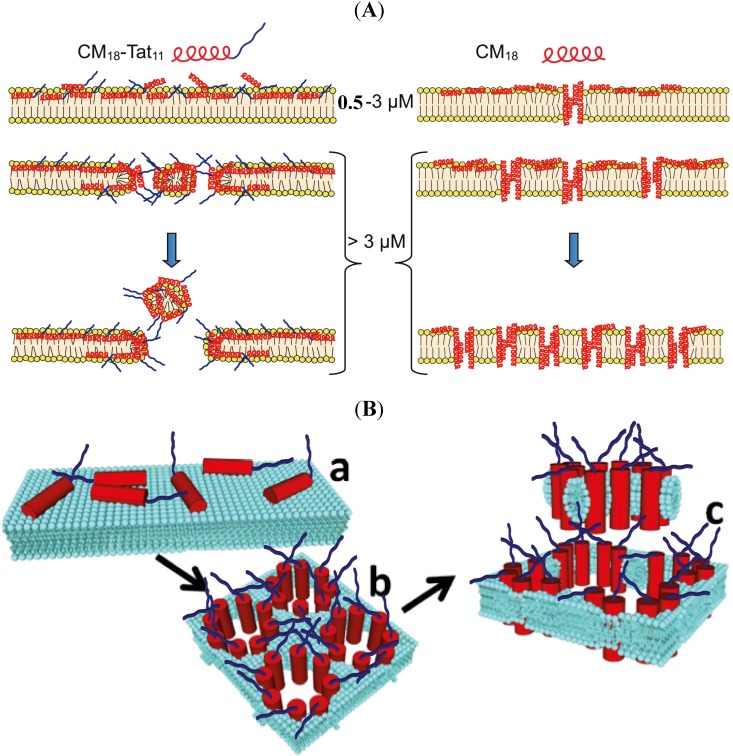
(**A**) Schematic representation of the membrane destabilization mechanism proposed for CM_18_-Tat_11_ (left) and CM_18_ (right) peptides. CM_18_ moiety is pictured as a red helix, while Tat_11_ moiety is represented as an unstructured blue segment. The upper part describes for each peptide its hypothetical membrane distribution when it is administered in the range between 0.5–3 µM. Instead the lower section represents the membrane distribution at concentrations higher than 4 µM. (**B**) Alternative mechanism of permeabilization operated by the CM_18_-Tat_11_ monomers, illustrated as red cylinders (CM_18_) plus blue segments (Tat_11_).

Based on these results, we conclude that the addition of the Tat_11_ sequence to the pore-forming CM_18_ module is determinant to trigger a switch in the membrane destabilization mechanism (schematic representation in Figure 5). The appearance of irreversible destabilization following an increase in CM_18_-Tat_11_ concentration from 3 to 4 µM seems to indicate a threshold-effect. This is somewhat surprising in light of the well-known ability of the Tat_11_ peptide to accumulate on the plasma membrane [1]. We believe this observation can be linked to the fact that the main constituents of the CHO-K1 extracellular matrix (e.g., heparan sulfates and membrane-associated proteoglycans, which are the electrostatic counterparts of Tat_11_ responsible for its accumulation on the plasma membrane [1]) are altered by the trypsinization procedure used here to detach the cells before the patch-clamp analysis. We wish to stress, however, the important result reported here, *i.e*., the demonstration of Tat_11_ ability to favor irreversible carpet-like membrane destabilization.

We believe this stems from the high positive charge density of Tat_11_, which leads to a stronger interaction of the peptide with the phospholipid head groups and to consequent membrane carpeting effect. However, it cannot be excluded that Tat_11_ may strongly promote membrane partitioning of CM_18_ (Figure 5B,a) up to a point that some of the resulting toroidal pores (characteristic of CM_18_ action) group together to delimit the contour of a micelle (Figure 5B,b), that may eventually separate from the membrane and pass in solution (Figure 5B,c).

## 3. Experimental

### 3.1. Peptide Synthesis and Purification

All peptides were prepared by solid-phase synthesis using Fmoc chemistry on an automatic Liberty-12-Channel Automated Peptide Synthesizer with an integrated microwave system (CEM, North Carolina, NC, USA). The crude peptides were purified by RP-HPLC on a Jupiter 4m Proteo 90 A column (250 × 10 mm; Phenomenex, Torrance, CA, USA). The HPLC analysis and purification was performed on a Dionex Ultimate 3000 PLC system (Sunnyvale, CA, USA) with autosampler. The correct purified product was confirmed by electrospray mass spectroscopy. The molecular weight of all peptides was confirmed by electrospray mass spectroscopy, and the concentration of each peptide stock solution was verified by UV-vis absorbance. The ESI-MS spectra of the peptides were obtained with an API3200QTRAP a Hybrid Triple Quadrupole/Linear Ion Trap (ABSciex, Foster City, CA, USA). The primary structure of CM_18_-Tat_11_ peptide was: acetyl- KWKLFKKIGAVLKVLTTGYGRKKRRQRRRC-CONH2; as a control, the single AMP and CPP moieties were also tested. All peptides were dissolved in bidistilled water to get a 500 μM stock solution stored at −80 °C. An aliquot of this stock was dissolved in the perfusion solution to get a specific final concentration and used within 30 min.

### 3.2. Cell Culture

Chinese Hamster Ovary (CHO-K1) cells were purchased from the American Type Culture Collection (CCL­61 ATCC) and were grown in Ham’s F12K medium supplemented with 10% fetal bovine serum at 37 °C and in 5% CO_2_, according to manufacturer’s instructions. For patch-clamp experiment cells were washed three times in PBS buffer and detached from plate by trypsination for 1 min in 0.25% trypsin solution (Invitrogen, Stockholm, Sweden). Cells were then centrifuged at 6,000 rpm for 1 min, and cell pellets dissolved in PBS for patch-clamp experiment.

### 3.3. Whole-Cell Patch-Clamp

Whole-cell recordings from CHO-K1 cells were obtained (by using an Axopatch 200B; Molecular Devices, Sunnyvale, CA, USA) under visual control at room temperature (20–22 °C). To faithfully record large currents (elicited by high concentrations of peptides and/or highly membrane permeabilizing ones), it is necessary to minimize *R_a_*, to reduce error in membrane potential control and time constant of charging the cell membrane capacitance. Moreover, the long tapered shank of the patch pipette may cause intracellular ion accumulation or depletion, and it slows down the rate of exogenous molecules incorporation via the patch pipette. These problems can be circumvented, all at once, by widening the patch pipette shank (Figure 1A) through the combination of heat (applied outside of the shank) and air pressure (applied to its lumen), as described previously [13]. Typical whole-cell recordings from CHO-K1 obtained with these pipettes had *R_a_* that ranged from 2 to 4 MΩ.

Peptides were applied and removed (in ~50 ms) by moving an automated multi-barrelled perfusion pipette on a horizontal plane in front of the recorded cell (Figure 1B). This enables us to rapidly switch the cell back and forth from a stream of control perfusion solution containing 130 mM of K^+^ (and 1 mM Ca^2+^ to preserve membrane integrity during long recordings) to a stream containing the tested peptide (dissolved in the same perfusion solution). Such an experimental strategy allows us to quantitatively describe membrane activity by measuring the kinetics of current change following both peptide application and withdrawal. Patch pipettes were filled with the perfusion solution to ensure that current is entirely driven by the holding potential (*V_h_*).

In the control solution, repetitive 10 mV pulses were routinely applied to check *R_a_* and *R_m_* stability (Figure 3). If there was a significant *R_a_* increase and/or *R_m_* decrease during a long control perfusion solution (*i.e*., there was a *R_a_* and/or an *R_m_* change larger than 1.5-fold), the recording was terminated. Recordings were also terminated if *R_m_* became <100 MΩ, for instance after the application of a peptide that yields irreversible membrane permeabilization (e.g., CM_18_-Tat_11_) or after many applications of a peptide that gives progressively larger permeabilization (e.g., CM_18_). The time lag between peptide application and the time when the current deviates from its baseline (following peptide application) more than three times of the noise average fluctuation, is referred as the *‘delay’* throughout the paper; during peptide application, current was considered at the steady state when its amplitude attained a value that did not change by more than 1% (an example is Figure 3B). Given the changes in the kinetics and amplitude of the current elicited by repetitive CM_18_ applications, the statistics were done considering the first peptide application only.

Recordings were filtered at 2 kHz via an eight-pole Butterworth filter (VBF/8 Kemo, Beckenham, UK), sampled on-line at 5 kHz by a Digidata 1322A (Molecular Devices) connected to the SCSI port of a Pentium computer running the pClamp 9.0 software package (Molecular Devices), and stored on disk.

Figures and statistics were performed using SigmaPlot (version 8.0; Jandel Scientific, San Rafael, CA, USA), and analyzed using Clampfit (version 9.0; Molecular Devices). All chemicals were purchased from Sigma (St. Louis, MO, USA). Results are given as means ± SEM.

## 4. Conclusions

The observations collected here allow us to readily explain the reported results on CM_18_-Tat_11_ as a delivery vector [1,2,3]. We shall argue that CM_18_-Tat_11_ enters the cell with no membrane perturbing effects at low (*i.e*., nanomolar) concentrations by means of the endocytic pathway, as already demonstrated [1]. Then, during the physiological vesicular trafficking, the peptide eventually reaches its critical membrane-perturbing concentration (≥4 µM), dissolves the bilayer integrity by a carpet mechanism, and promotes the release of the co-administered macromolecules that specifically target the same route. An irreversible membrane destabilization accounts for the observed vesicle release of macromolecules with hydrodynamic radii up to 100 nm (as for plasmidic DNA), a value considerably larger than the openings of the transmembrane toroidal pores (~1–10 nm [7]).
